# Characteristics of the Gut Microbiota and Potential Effects of Probiotic Supplements in Individuals with Type 2 Diabetes *mellitus*

**DOI:** 10.3390/foods10112528

**Published:** 2021-10-21

**Authors:** Rafael Ballan, Susana Marta Isay Saad

**Affiliations:** 1 Department of Pharmaceutical and Biochemical Technology, School of Pharmaceutical Sciences, University of São Paulo, São Paulo 05508-000, SP, Brazil; Rafael.maluhy@gmail.com; 2 Food Research Center, University of São Paulo, São Paulo 05508-080, SP, Brazil

**Keywords:** diabetes *mellitus* type 2, gut microbiota, microbial metabolites, dysbiosis, probiotics

## Abstract

The increasing prevalence of type 2 diabetes *mellitus* (T2DM) worldwide has become a burden to healthcare systems. In 2019, around 463 million adults were living with diabetes *mellitus*, and T2DM accounted for 90 to 95% of cases. The relationship between the gut microbiota and T2DM has been explored with the advent of metagenomic techniques. Genome-wide association studies evaluating the microbiota of these individuals have pointed to taxonomic, functional, and microbial metabolite imbalances and represent a potential intervention in T2DM management. Several microbial metabolites and components, such as imidazole propionate, trimethylamine, and lipopolysaccharides, appear to impair insulin signaling, while short-chain fatty acids, secondary bile acids, and tryptophan metabolites may improve it. In addition, the use of probiotics with the aim of transiently restoring the microbial balance or reducing the effects of microbial metabolites that impair insulin sensitivity has been explored. Herein, we critically review the available literature on the changes in the gut microbiota in T2DM together with potential adjuvant therapies that may improve the health status of this population.

## 1. Introduction

Diabetes *mellitus* (DM) describes a group of metabolic disorders and is mainly characterized by chronic hyperglycemia resulting from impaired insulin secretion or impaired insulin action or both mechanisms together, causing long-term complications [1]. Persistent hyperglycemia is associated with chronic micro and macrovascular complications. People with diabetes are at an increased risk of developing numerous health problems that may be life threatening, such as vascular damage that affects the heart, eyes, kidneys, and nerves [2]. Clinical presentation and the progression of type 1 and type 2 diabetes usually vary considerably due to the distinct pathophysiology of the diseases. An early accurate classification is important for determining therapy although this is sometimes is not possible [3].

Type 2 diabetes (T2DM), which accounts for 90 to 95% of all DM cases, is the most common metabolic disorder and is characterized by insulin resistance and pancreatic β-cell dysfunction, which lead to unstable hyperglycemia [4,5]. In the case of β-cell dysfunction, insulin secretion is decreased, limiting the body’s ability to maintain physiological plasma glucose levels, whereas insulin resistance contributes to increased glucose production in the liver and decreased glucose uptake in the muscle, adipose tissue, and liver [6]. It has a complex and multifactorial etiology that involves genetic and environmental components and usually affects individuals from the fourth decade of life, although there has been an increase of the incidence of diabetes in children and young people. The risk factors for T2DM are genetic susceptibility, age, obesity, physical inactivity, previous diagnosis of pre-diabetes or gestational diabetes (DMG), inadequate diet, and stress [2,7].

According to the Diabetes Atlas of the International Diabetes Federation (IDF) 2019 [8], approximately 463 million adults (20–79 years) in the world, corresponding to 9.3% of the world population, are living with diabetes; it is estimated that this number will increase to 700 million in 2045. In 2019, 374 million people were at risk of developing T2DM, and this proportion has increased in many countries. The largest number of people with diabetes are between 40- and 59-years-old. For every two people with diabetes, one does not know that they have the disease, or 263 million people.

The most probable explanations for the increased prevalence of diabetes are social and economic changes, including changes towards a sedentary lifestyle, an unbalanced diet leading to the worsening of nutritional status, an increased prevalence of being overweight, and growing urbanization. On the other hand, improved health care has increased the life expectancy of people with diabetes. Another explanation for the increase in diabetes prevalence is the availability of more recent data since the disease was previously underreported [9].

In general, T2DM is associated with elevated levels of pro-inflammatory cytokines, chemokines, and inflammatory proteins. Patients with T2DM usually have a high-fat diet that is associated with increased lipopolysaccharide production by Gram-negative bacteria in the gut, and its passage to the blood circulation triggers inflammatory responses that lead to insulin resistance [6]. Both genetic and epigenetic factors have been implicated in the development of inflammation associated with T2DM. The dysregulation of the epigenetic control mechanisms that control the expression of a great number of genes has been linked to the pathogenesis of several disorders related to the immune system, including T2DM. It is well established that the presence of a pro-inflammatory phenotype is strongly associated with the development of insulin resistance, β cells, and vascular complications in a patient with DM [10]. Hyperglycemia and dyslipidemia cause abnormal epigenetic changes that promote the activation of the main inflammatory pathways and that contribute to the development of a state of low-grade chronic inflammation in T2DM. This state of chronic inflammation impairs insulin secretion and sensitivity, leading to the development of T2DM and its comorbidities [4]. The development and perpetuation of hyperglycemia occur concomitantly with hyperglucagonemia; the resistance of peripheral tissues to the action of insulin; increased hepatic insulin resistance, incretin dysfunction, increased lipolysis, and a consequent increase in free circulating fatty acids; increased renal reabsorption of glucose; and varying degrees of deficiency in the synthesis and insulin secretion by the pancreatic β cell. T2DM may be controlled through measures such as changes in lifestyle and the adoption of healthier diets in association with medications, if necessary [7].

Pharmaceutical treatments used to treat this condition involve several classes of drugs, which act to reduce glucose production and absorption, increase insulin production and response, or increase urine glucose excretion. The standard drugs used to treat T2DM are metformin and glucagon-like peptide 1 (GLP-1) receptor agonists [3]. However, there are limitations in treatment with medications, as some patients are allergic to these medications or have serious adverse effects such as diarrhea and lactic acidosis [11,12].

The gut microbiota plays an important metabolic role, either through its ability to ferment non-digestible carbohydrates and to synthesize micronutrients or through its interaction with the immune system [13]. The term microbiota refers to the assemblage of living microorganisms, including bacteria, archaea, protozoa, fungi, and algae, that is present in a defined environment [14]. Recently, changes in the human intestinal microbiota have been associated with pathological conditions such as obesity and other metabolic disorders such as type 2 diabetes mellitus, metabolic syndrome, and insulin resistance [15,16]. Among the mechanisms that associate the intestinal microbiota with diabetes and insulin resistance, there is an increase in the permeability of the intestinal barrier, resulting in metabolic endotoxemia. In addition, an increased production of branched-chain amino acids (BCAA), imidazole propionate, and of trimethylamine N-oxide (TMAO) as well as interaction with bile acids, changes in fatty acid metabolism, and intestinal hormones also occur. These changes may lead to increased levels of adiposity and impaired insulin signaling [15,17,18].

The first-line drug for managing T2DM, metformin, has already been linked to an increase in the abundance of *Escherichia*, *Intestinibacter*, *B*. *adolescentis*, and *Akkermansia muciniphila*. An increase in short chain fatty acid (SCFA) production was also observed, suggesting that the modulation of the gut microbiota mediates some of the antidiabetic effects of metformin [19].

Since T2DM represents the variation of the disease that affects the most diabetic patients and that is frequently associated with obesity and cardiovascular diseases, efforts have been made to develop new therapies to control and prevent the disease [20].

With the development of the metagenomics techniques, the whole-genome sequencing of all the DNA contained in a sample as well as a taxonomic investigation at the species and strain level became possible, providing a functional profile of the metabolic pathways present in a community. With these features, a better understanding of the relationship between the gut microbiota and T2DM should lead to advances in therapeutic approaches and the development of new therapies, such as the use of probiotics.

Herein, we critically summarize recent findings on the role of the microbiota in T2DM as well as the use of probiotic supplements in the metabolic parameters of individuals with T2DM.

## 2. Materials and Methods

### 2.1. Search Method

The integrative review was performed using the following terms: type 2 diabetes mellitus AND (microbiota OR microbiome OR probiotics OR *Lactobacillus* OR *Bifidobacterium* OR *Akkermansia*). Trials were identified by searching the MEDLINE (via Pubmed), Scopus, and Web of Science databases in the period between 2011 and March 2021. We included human studies published in English, Spanish, or Portuguese. Studies evaluating whether an intervention with a probiotic supplement compared to treatment with a placebo had any effect on at least one parameter related to the glucose profile (e.g., hemoglobin A1C, fasting plasma glucose, or insulin levels) were included. To explore the association between gut microbiota and type 2 diabetes mellitus, studies evaluating gut microbiota or functional changes in type 2 diabetes mellitus were included.

### 2.2. Eligibility Criteria and Study Selection

Duplicates were removed manually. For the association between gut microbiota and type 2 diabetes *mellitus*, cohorts or case–control studies evaluating microbial composition by means of genetic sequencing (16S rRNA or metagenomic sequencing) were included. For the assessment of supplementation with probiotics, randomized controlled trials in which probiotics in the form of any pharmaceutical formulation administered to adult patients with T2DM were included after title and abstract screening. Combination therapy (e.g., minerals, prebiotics, fatty acids or phytosterols) or associated diseases were the exclusion criteria. Subsequently, the full texts of the articles were reviewed for the inclusion of eligible studies.

**Figure 1:**
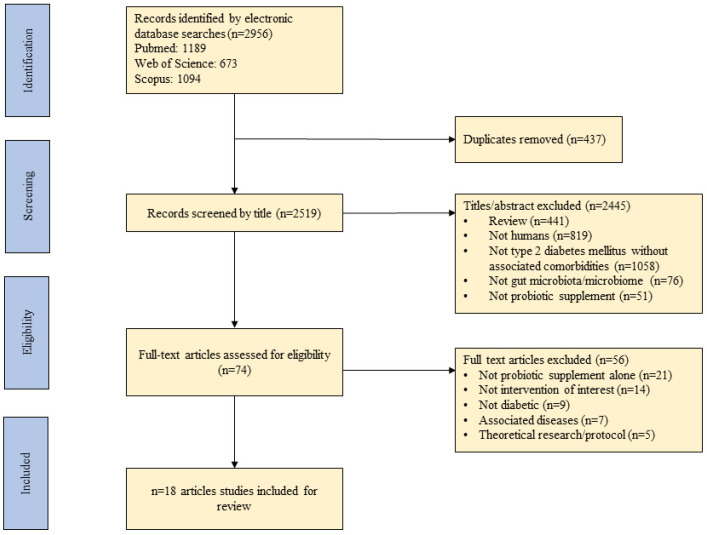
A flow diagram detailing the process followed for the selection of the studies for the integrative review.

Figure 1 outlines the steps followed for the selection of the studies included in this review.

## 3. Gut Microbiota and Association with T2DM

Several studies suggest that the susceptibility, development, and progression of T2DM is influenced by the gut microbiota [21,22,23,24]. This is due to a reduction in diversity and a microbial imbalance, leading to an impact on the immune system and the emergence and growth of pathogens. Dysbiosis is also associated with obesity, insulin resistance, and low-grade inflammation, which reflects a possible causality linking these pathologies [24].

Several human studies have reported bacterial genera or species that are reduced or increased in T2DM patients compared to healthy controls. A summary of recent studies evaluating changes in the gut microbiota found in T2DM is shown in Table 1.

The idea that the microbiota might contribute to the development of diseases such as obesity comes from animal studies conducted in the early 2000s. Among the main findings are the increased Firmicutes/Bacteroidetes ratio in obese patients and the association of microbiota with obesity [31,32]. By transferring the gut microbiota from obese to germ-free mice, their energy harvest capacity and adiposity were increased, establishing a possible causal relationship.

In the following years, several other studies reported an enrichment or depletion of bacterial genera or species in the gut microbiota, indicating a connection with adiposity, insulin resistance, and T2DM. The first study to describe differences in the microbial composition between healthy individuals and individuals with T2DM dates from 2010 [33]. Thirty-six stool samples were evaluated using 16S rRNA amplicon sequencing between individuals with T2DM and healthy controls. T2DM was associated with dysbiosis at the phylum level, with a reduction in the proportion of Firmicutes and an increase in *Bacteroidetes* and *Proteobacteria*, while the Chao1 diversity of the gut microbiota was positively correlated with body mass index (BMI) in T2DM patients [33]. On the other hand, these results were not observed in two large-scale metagenome-wide association studies performed in China and Europe [23,25]. Furthermore, the European study found the genus former-*Lactobacillus* as discriminant for T2DM, while the Chinese study did not.

Controversial results also occurred for the species *Akkermansia muciniphila*. In a study conducted with 368 Chinese subjects, *A*. *muciniphila* was found to be increased in T2DM, while in another two studies with Chinese subjects, a reduction in abundance was found in T2DM. Animal studies systematically report that *A*. *muciniphila* abundance is inversely correlated with body weight, fat mass, insulin resistance, glucose intolerance, and inflammation [34,35,36,37]. In a randomized, double-blind, placebo-controlled clinical trial conducted with overweight or obese insulin-resistant patients, the supplementation of pasteurized *A*. *muciniphila* resulted in weight loss, improved insulin resistance, reduced insulinemia, and plasma total cholesterol. However, it is important to emphasize that this was only a pilot study to verify the safety and tolerance of its supplementation (*n* = 32) [38]. A potential mechanism suggested for these positive effects is an interaction between the thermostable outer membrane protein Amuc 1100, which is found in pasteurized *A*. *muciniphila*, and with Toll-like receptor 2 [34].

Overall, patients with TDM2 have a reduced abundance of SCFA producing species (*Faecalibacterium prausnitzii*, *Roseburia intestinalis*, *R*. *inulinivorans*, *R*. *faecis*, *Akkermansia muciniphila*, *Bifidobacterium* spp., and *Eubacterium rectale*) and tryptophan metabolite producing bacteria (*Bifidobacterium*, former-*Lactobacillus*, *Ruminococcus*, *Bacteroides*, and *Clostridium*). On the other hand, these patients have an increased abundance of opportunistic pathogens (*Clostridium hathewayi*, *Clostridium ramosum*, *Bacteroides caccae*, *Escherichia coli*, and *Eggerthella lenta*), sulfate-reducing bacteria (*Desulfovibrio* spp.), and branched-chain amino acid producing bacteria (*Prevotella copri*, and *Bacteroides vulgatus*) compared to healthy controls [21,22,23,25,26,27,28,29,30].

At the functional level, the main pathways enriched in T2DM were the metabolism of BCAAs and fatty acid biosynthesis and transport, while the bacterial methanogenesis and metabolism of cofactors and vitamins were depleted [22,23,25,27,29]. BCAA concentrations are known to correlate positively with insulin resistance, and *Prevotella copri* was found to be the major driving species between microbial biosynthesis in the gut and insulin resistance, suggesting a potential causal relation that deserves better investigation [27]. A Chinese and a Swedish study also found an increased expression of the microbial genes involved in oxidative stress, suggesting that the gut microbiota in T2DM stimulates bacterial defense mechanisms against the oxidative stress characteristic of the disease [23,25].

Despite the similarities found in these studies, some of the conflicting results must be explained by differences in geographic locations, genetics, drug treatment, and sequencing techniques. The development of novel technologies is highly desirable to understand whether these associations between gut microbiota and T2DM are causal or if they are consequences of the development of the disease.

## 4. Microbial Metabolites and Components Linked to T2DM

In recent years, low-grade inflammation has been hypothesized to be the link between the microbiome and the risk for developing T2DM due to mechanisms related to microbial metabolites such as bacterial toxins, short-chain fatty acids, bile acids, TMAO, tryptophan metabolites, and BCAA metabolites. Microbial metabolites allow us to better understand the underlying mechanisms by which bacterial taxa contribute to host health and disease. The main microbial metabolites related to T2DM are shown in Table 2.

### 4.1. Low-Grade Inflammation

Metabolic diseases, such as obesity and T2DM, share a characteristic: the chronic state of low-grade inflammation. The proposed mechanism in the literature is the activation of Toll-like receptors (TLR) by the lipopolysaccharides (LPS) that are present in the cell wall of Gram-negative bacteria. LPS is an endotoxin that leads to a chronic systemic inflammatory response when it is consistently increased in serum levels. This situation occurs in T2DM due to exacerbated bacterial translocation [49,50].

TLRs, on the other hand, comprise a large family of cellular membrane proteins that play a crucial role in the innate immune response, providing the first line of defense against host pathogens. Through the recognition of pathogen-associated molecular patterns (PAMPs), TLRs activate cascades signaled by inflammatory cytokines in the target tissues of insulin. This, in turn, leads to the activation of the phosphorylation of kinases, c-Jun n-Terminal, and IκB, increasing the inflammatory response [51]. The result of this sequence of molecular events is the inhibition of the insulin transducer signal via the phosphorylation of insulin receptor substrate 1 (IRS1) in serine, leading to insulin resistance in the liver, adipose, and muscle tissues. This mechanism inhibits the signaling of the insulin receptor tyrosine kinase and protein kinase b (AKT), contributing to the degradation of IRS-1 and towards insulin resistance [52,53].

### 4.2. Short-Chain Fatty Acids

T2DM patients are known to have a reduced abundance of SCFA-producing bacteria, such as the *Roseburia*, *Eubacterium*, *Faecalibacterium,* and *Ruminococcus* species [21,23]. This leads to a reduction in the production of the SCFAs acetate, butyrate, and propionate in the host colon, which is derived from the fermentation of non-digestible carbohydrates. Among their functions, SCFAs play a role in the cell growth and differentiation, in the maintenance of intestinal epithelial integrity, and in immunomodulatory and anti-inflammatory effects [39,40]. By binding to G-protein-coupled receptors 41 and 43, SCFAs stimulate the production of GLP-1 peptides from colonic enteroendocrine L cells and peptide YY (PYY) [39]. These peptides reduce gastric emptying, control the appetite, stimulate insulin secretion, and inhibit glucagon secretion. In a clinical trial, the increase in SCFA levels promoted by dietary fiber intake was associated with lower levels of HbA1c, which was partially due to the increase in GLP-1. The authors concluded that recovering active SCFA-producing bacteria may alleviate T2DM phenotypes and represent a new way to manipulate the microbiota in T2DM and other dysbiosis-related diseases [54].

### 4.3. Trimethylamine N-Oxide

Trimethylamine (TMA) is an organic compound synthesized by the microbial metabolism of dietary phosphatidylcholine, choline, and carnitine. After being absorbed, it is transported to the liver through the portal vein. TMA is converted in the liver by flavin monooxygenase 3 (FMO3) to form TMAO [55].

Several strains of bacteria are potentially TMA/TMAO producers in vivo, including Firmicutes, Proteobacteria, *Anaerococcus hydrogenalis*, *Clostridium asparagiforme*, *C*. *hathewayi*, *C*. *sporogenes*, *Escherichia fergusonii*, *Proteus penneri*, *Providencia rettgeri*, *Edwardsiella tarda*, and *Desulfuricanibrio desulfuricans* [43,56].

T2DM patients have increased TMAO serum levels. Higher TMAO plasma levels are associated with an increased risk of T2DM, cardiac events, and mortality [57]. In animals, TMAO intake has been associated with worsening impaired glucose tolerance and insulin resistance induced by a high fat diet (HFD). This is also mediated by the insulin signaling pathway in the liver, increasing the production of inflammatory cytokines in the adipose tissue [58].

In contrast, a recent Mendelian randomization found that T2DM elevates TMAO levels, suggesting reverse causality. Therefore, the relationship between T2DM and TMAO requires more investigation to elucidate the issue [59].

### 4.4. Imidazole Propionate

Imidazole propionate (ImP) is a microbially produced histidine-derived metabolite. Bacteria usually produce ImP from its precursor urocanate. Potentially, ImP producers are *Eggerthella lenta*, *Streptococcus mutans*, *Aerococcus urinae*, *Brevibacillus laterosporus*, and others. Patients with T2DM show an increase in Imp-producing bacteria in addition to having increased levels in the portal vein and peripheral blood [42,60]. Administration of ImP in mice have demonstrated that it impairs glucose tolerance. Consistent with the findings in mice, in the human liver, the phosphorilation of p62 and S6K1 was higher than it was in healthy controls, indicating a role for imidazole propionate in impairing insulin signaling through the p62 and mTORC1 pathway [42].

ImP represents an important target for the development of approaches that modify its production from bacteria, resulting in an improvement in insulin resistance.

### 4.5. Branched-Chain Amino Acids

Branched-chain amino acids (BCAAs), which include leucine, isoleucine, and valine, are essential amino acids. Former-*Lactobacillus*, *Weissella,* and *Leuconostoc* are among the genera capable of producing BCAAs. In addition, *Prevotella copri* and *Bacteroides vulgatus* are known as the main species driving the association between biosynthesis of BCAAs and insulin resistance [45]. Despite this, several studies have highlighted that the metabolism of microbial amino acids may play a role in the development of insulin resistance. Human studies have shown that the increased intake of BCAAs is associated with an increased risk of insulin resistance and T2DM [61]. This may be due to the insulinogenic activity of these amino acids. Constant high levels of BCAAs persistently activate the mTORC1 signaling pathway, leading to IR with the serine phosphorylation of insulin receptor substrate 1 (IRS-1), which occurs in response to persistent aminoacidemia or hyperinsulinemia [62,63]. Among BCAAs, leucine seems to be more important in this process, as it has a greater effect on the mediation of mTORC1 activity [62]. In contrast, BCAAs were associated with a lower risk of developing T2DM in Japanese women [64]. The increase in insulin resistance caused by the increased plasma levels of BCAAs appears to be context-dependent, and it is possible that BCAAs play different roles in glucose metabolism among persons with IR conditions. The role of BCAAs in the development of T2DM is still controversial and requires further investigation.

### 4.6. Tryptophan Metabolites

Tryptophan is one of the nine essential amino acids, and because it is not synthesized by the human body, it needs to be supplied by the diet. Tryptophan is absorbed in the small intestine, but the fraction that reaches the colon can be catabolized by the gut bacteria in several indole-derivatives. Many bacterial species are able to catabolize tryptophan. They belong to the genera *Bacteroides*, *Clostridium*, *Bifidobacterium*, former-*Lactobacillus*, *Anaerostipes*, among others, and produce the metabolites Indole, 3-methylindole (Skatole), indoleacetic acid (IAA), indoleacrylic acid (IA), indolealdehyde (IAId), indolelactic acid (ILA), indolepropionic acid (IPA), and tryptamine [48].

**Figure 2:**
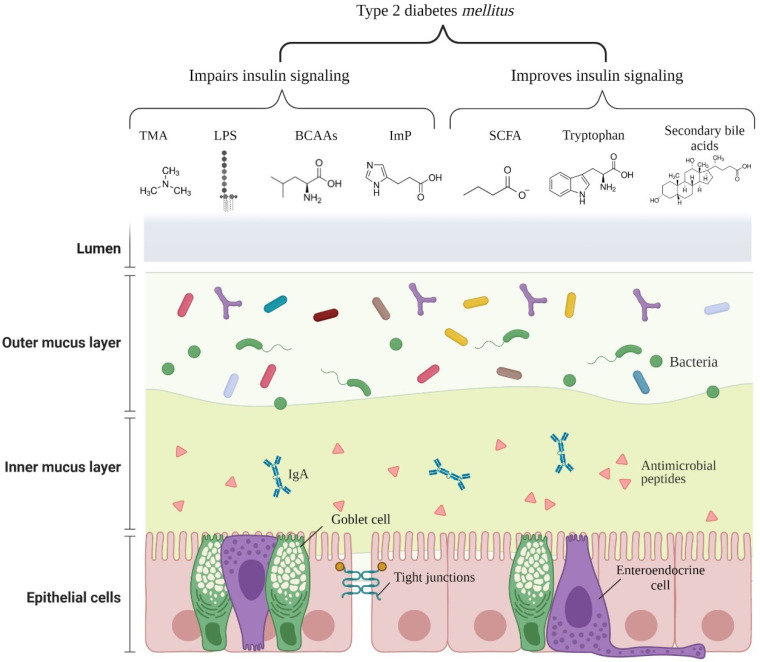
Main microbial components/metabolites affecting insulin signaling in type 2 diabetes *mellitus*. Abbreviations: TMA: trimethylamine; LPS: lipopolysaccharide; BCAAs: branched-chain amino acids; ImP: imidazole propionate; SCFA: short chain fatty acids; IgA: immunoglobulin A. (Get permission from BioRender. 18 October 2021).

Figure 2 illustrates the main microbial components and metabolites affecting insulin signaling.

In mice, IPA was able to regulate the intestinal barrier function by acting as a pregnane X receptor (PXR) ligand, reducing the intestinal permeability in mice fed with HFD [65,66]. IA was also able to promote intestinal epithelial barrier function and reduce inflammatory response in mice by mucus production and as well as to promote goblet cell differentiation [67]. Higher serum IPA levels were associated with a reduced risk of developing T2DM and better insulin secretion, possibly through the preservation of β-cell function [68].

Additionally, indole was able to induce GLP-1 secretion in mouse colonic enteroendocrine L cells, suggesting that indoles might play an important role in the glucose metabolism [69]. An increase in GLP-1 levels stimulates insulin production, reduces appetite, and delays gastric emptying, which could benefit T2DM patients. Human studies endorsing these effects are desirable and should be performed in the future [70].

### 4.7. Bile Acids

Bile acids are produced and secreted by the liver and are then released into the intestine. Secreted bile acids are reabsorbed in the intestine, mostly in the ileum. In the ileum and colon, the gut bacterial bile salt hydrolase (BSH) converts primary conjugated bile salts into deconjugated bile acids (BA) that are subsequently converted into secondary BA [47]. BSH activity is high in bacteria belonging to the genera *Clostridium*, *Bacteroides*, *Bifidobacterium*, and former-*Lactobacillus* [46]. Gut bacteria regulates bile acid composition and pool size to modulate the intestinal farnesoid X receptor (FXR) and Takeda G protein-coupled receptor 5 (TGR5) signaling. While the primary bile acids activate FXR, secondary bile acids bind to the G protein-coupled TGR5 receptor, which results in GLP-1 secretion in enteroendocrine L cells [47]. This mechanism has been demonstrated in obese diabetic mice, where intestinal FXR activation altered bile acid metabolism by increasing lithocholic acid (LCA)-producing bacteria in the gut [71]. Higher LCA levels activated intestinal TGR5, which then stimulate GLP-1 to improve hepatic glucose and lipid metabolism. This mechanism represents a potential therapeutic target for the treatment of T2DM and other metabolic diseases.

## 5. Effects of Probiotics Supplements in T2DM

Probiotics are defined as live microorganisms that, when administered in an adequate amount, confer a health benefit on the host [72]. Controversies about the effects of probiotics have generated repercussions among researchers, and some studies warn about the potential risks of their use [73]. However, several benefits of taking probiotics have been reported, including immunomodulation, SCFAs production, antagonism with pathogens, improvement of the barrier function, gut microbiota modulation, enzyme production, and the production of small molecules, with systemic effects [74].

Currently, most clinical trials mainly focus on probiotics of the former-*Lactobacillus* and *Bifidobacterium* genera. However, with the advances in microbiota research, single bacterial strains associated with the improvement of inflammation-related diseases are screened and isolated. Some of these strains are expected to emerge as next generation probiotics [75].

In the search for new approaches to control T2DM, the number of experiments using probiotics to improve the glycemic and lipid profile in T2DM patients is rising, but the number is still low. However, it is necessary to exercise caution when evaluating these studies, as it is known that the effect of probiotics depends on the strain that is chosen, the characteristics of the group that is being studied, the pathophysiology of the disease, the food matrix or pharmaceutical form, whether it is single or multi-strain, the intervention period, and the sufficient dose [76].

In this review, we have focused on studies exclusively using probiotic supplements without other compounds or substances, such as fatty acids, vitamins, and minerals, to avoid confounding factors. A summary of the clinical trials is shown in Table 3.

Sabico et al. evaluated the use of 10^10^ CFU/day of a multi-strain probiotic preparation containing *Bifidobacterium bifidum* W23, *Bifidobacterium lactis* W52, *Lactobacillus acidophilus* W37, *Levilactobacillus brevis—*former *Lactobacillus brevis—*W63, *Lacticaseibacillus* *casei—*former *Lactobacillus casei—*W56, *Ligilactobacillus* salivarius—former *Lactobacillus salivarius—*W24, *Lactobacillus lactis* W19, and *Lactobacillus lactis* W58) regarding metabolic endotoxemia levels and cardiometabolic parameters in adult patients recently diagnosed with T2DM, for 12 weeks. A clinically significant improvement in HOMA-IR (homeostatic model assessment-insulin resistance) and a reduction in the waist–hip ratio between groups were observed. Within-group comparisons in the probiotic group resulted in lower levels of fasting blood glucose (FBG), insulin, insulin resistance, C-peptide, triglycerides, and LDL-c. No significant changes in endotoxin levels were observed [79]. In another study, the effects of the same commercial probiotic preparation, in the same amount and considering the same parameters as the previous study, were evaluated for a period of 6 months. Again, a clinically significant difference was observed in the HOMA-IR, and a borderline significant improvement in the insulin levels in the probiotic group was also seen. Within-group comparisons showed a reduction in the inflammatory markers (TNF-α, IL-6, C-reactive protein) and an improvement in the endotoxin and adiponectin levels. The results obtained were satisfactory, corroborating the idea that the duration of probiotic supplementation is also essential for its action [83]. In agreement with these results, the same product was shown to be able to improve the intestinal epithelial barrier function in vitro [86].

Integrity of the intestinal barrier has been one of the main focuses of improvement with the use of probiotics. A reduction in low-grade inflammation and improvements in the insulin signaling cascade are expected when the passage of LPS is reduced. In this context, Karczewski et al. elegantly evaluated the impact of the probiotic *Lactiplantibacillus plantarum* (former *Lactobacillus plantarum*) strain WCFS1 when it was injected directly into the duodenum of a group of individuals followed by a tissue biopsy 6 h after the intervention. The authors observed increased translocation of *zonula occludens*-1 and occludin close to the tight junctions, promoting intestinal epithelium integrity in a TLR2-dependent manner [87]. Similar results were obtained in cell cultures for several strains of the former-*Lactobacillus* genus [88].

In a 9-month double-blinded, randomized, placebo-controlled study, Hsieh et al. observed a decrease in the serum levels of HbA1C and cholesterol in patients with T2DM who received capsules containing probiotic *Limosilactobacillus reuteri* (former *Lactobacillus reuteri*) ADR-1 [81]. The reduction in HbA1C remained even after three months of follow-up without probiotic intake. Microbiota analysis revealed increased levels of *L.* *reuteri* in the probiotic group and that changes in HbA1C were negatively correlated with the upregulation of the total *L.* *reuteri* and that it was positively correlated with the *Bacteroidetes* or *Bacteroidetes*/*Firmicutes* ratio. Together, these results indicate that the degree of HbA1C reduction is affected by the upregulation level of *L.* *reuteri* in T2DM after *L.* *reuteri* ADR-1 consumption [81].

Similarly, an RCT conducted by Mobini et al. evaluated the intake of *L.* *reuteri* DSM 17,938 at different concentrations (low dose: 10^8^ CFU/day vs. high dose: 10^10^ CFU/day) for 12 weeks but did not observe a reduction in HbA1C in T2DM patients. An improvement in the insulin sensitivity index (ISI) was observed in the high dose group even though it was not significant. However, post hoc analysis based on ISI improvement showed a significant reduction in HbA1C and secondary bile acids after *L.* *reuteri* intake and evidenced that responders had higher microbiota diversity at baseline [80].

A 12-week intervention with a probiotic multi-strain (3 × 10^10^ CFU/day of *L*. *acidophilus*, *L*. *casei*, *L. lactis*, *B. bifidum*, *B*. *longum,* and *B*. *infantis*) with 101 T2DM adults was able to reduce HbA1C levels, fasting insulin, and insulin resistance and demonstrated an increase in *Bifidobacterium* and former-*Lactobacillus* species in the gut microbiota of the probiotic group [78].

An RCT conducted by Palacios et al. evaluated the effect of a probiotic multi-strain (2 × 10^11^ CFU/day, containing *L*. *plantarum* Lp-115, *L*. *bulgaricus* Lb-64, *L*. *gasseri* Lg-36, *B*. *breve* Bb-03, B. *animalis* sbsp. *lactis* Bi-07, *B*. *bifidum* Bb-06, *Streptococcus thermophilus* St-21, and *Saccharomyces boulardii* DBVPG 6763) in patients with prediabetes and T2DM [85]. Only an increase in the butyrate plasma levels was found between the intervention and the placebo groups. Interestingly, the sub-group analysis in participants taking both the metformin and probiotic showed reductions in their levels of FBG, HbA1c, insulin resistance, and zonulin, a marker and modulator of the intestinal permeability. The authors hypothesized that probiotics and metformin altered zonulin levels, encouraging the microbiome to increase luminal butyrate production. Microbiota analysis revealed that either the probiotic alone or in combination with metformin was able to increase SCFA-producing bacteria after 12 weeks of intervention [85].

Among the nine clinical trials that were selected here, three of them were of short duration (8 weeks or less). Mazloom et al. did not find significant differences in anthropometric and metabolic parameters with the use of probiotics for 6 weeks in T2DM [77]. A reduction in FBG, insulin concentration, and insulin resistance along with an increase in SIRT1 and a decrease of fetuin-A was observed by Khalili et al. in a study using a single-strain probiotic (10^8^ CFU/day of *L*. *casei*) for 8 weeks [84]. Additionally, Kobyliak et al. found a reduction in the HOMA-IR and inflammatory markers (TNF-α, IL-1β) when administering a multi-strain probiotic (14 probiotic bacteria genera *Bifidobacterium,* former-*Lactobacillus, Lactococcus, Propionibacterium*) for 8 weeks [82].

Searching for probiotic effects under altered parameters in individuals with T2DM was a challenge due to the scarcity of the literature, inappropriate experimental designs, and high heterogeneity in the form of administration. In a recent meta-analysis that only included randomized clinical trials conducted with T2DM, Kocsis et al. found that the probiotics were able to improve fasting blood glucose (−16.52 mg/dL, 95% CI—23.28; −9.76, *p* < 0.001), HbA1c (−0.33%, 95% CI—0.53; −0.13, *p* = 0.001), and fasting insulin (1.40 μIU/mL, 95% CI—2.52, −0.27, *p* = 0.015) [89]. However, probiotics in foods and therapies combined with other substances were considered. Additionally, in the subgroup analysis, no significant improvement was observed with long-term interventions, higher doses, or the use of multi-strain probiotics, although other studies have found an improvement [76,90,91].

We must consider that the application of probiotics is strain-specific and disease-specific and that grouping the results obtained with different strains for different outcomes may lead to inaccurate conclusions. Often, only the genus or genus and species are reported in studies, making comparison difficult. Here, only two studies used the same probiotic formulation as an intervention and obtained positive results, mainly for insulin resistance [79,83]. Two studies evaluated strains of *Limosilactobacillus* *reuteri,* and one study evaluated a strain of *Lacticaseibacillus casei*, which also resulted in benefits in terms of the glucose metabolism in T2DM patients [80,81,84]. Two other RCTs that were included identified that the gut microbiota composition seemed to be decisive in obtaining a response with the intervention of probiotics [80,81]. Only one study evaluated the microbiota at the functional level, obtaining results that do not resemble those observed in the cohorts dedicated to evaluating these alterations [85].

Overall, further well-controlled RCTs with strains or formulations that have proven effectivity should be performed to improve the level of evidence that is currently available. Furthermore, the administration of species that are inversely correlated with diabetes markers, such as *Akkermansia muciniphila*, should be conducted with the possibility of obtaining better results. Finally, evaluating changes at the functional level of the gut microbiota is also desired to elucidate the way in which probiotics modify microbial metabolism and impact the host.

## 6. Conclusions and Future Perspectives

Despite the substantial increase in publications evaluating the effect of probiotics on T2DM, the methodologies are highly heterogeneous, usually lack adequate control, and are often not comparable. This review was dedicated towards evaluating studies using only probiotic supplements to avoid the confounding effects caused by food matrices and other substances in association. It is known that the probiotic effects are strain-specific and disease-specific, and in future clinical trials dedicated to evaluating the effect of the most promising strains should be conducted, thereby allowing a better comparison between them.

Many of the bacterial species altered in the T2DM gut microbiota are not cultivable, which represents a challenge for investigating the effects of their supplementation. Despite this, a pilot clinical trial has already been developed to verify their safety and tolerance with *Akkermansia muciniphila*, a bacterium that is difficult to culture due to its sensitivity to oxygen. This fact leads to the possibility that new technological and cultivation techniques may allow for the cultivation of strictly anaerobic strains from the host gut microbiota. Together, SCFA-producing bacteria supplementation should be performed due to its low abundance in the T2DM gut microbiota.

The association of changes in the gut microbiota, both at the compositional and functional levels, and T2DM traits is well documented. Together with microbial metabolites, they play a critical role in the etiology of T2DM. Future research aiming not only at taxonomic changes in the microbiota but also at altered metabolic pathways in T2DM, should be performed in human clinical trials, with the possibility of obtaining effective and comparative results. Current literature indicates that some strains appear to have a beneficial effect in patients with T2DM, but further studies are needed to support the use of these probiotics as an adjuvant therapy in the management of T2DM.

## Figures and Tables

**Table 1:** Summary of studies evaluating microbial and functional changes in individuals with T2DM.

Sample Size	Age (y)	Sex	Technique	Microbiota Modifications	Functional Modifications	Study
183 T2D 185 Controls (Chinese)	13–86	Women (153) Men (209)	Metagenomic sequencing	Increased in T2D: *A*. *muciniphila*, *Bacteroides caccae*, *Bacteroides Intestinalis*, *C*. *hathewayi*, *Clostridium ramosum*, *C*. *symbiosum*, *Desulfovibrio* sp., *Eggerthella lenta*, and *Escherichia coli* Decreased in T2D: *Clostridiales* sp. *SS3/4*, *Eubacterium rectale*, *F*. *prausnitzii*, *Roseburia intestinalis*, and *Roseburia inulinivorans.*	Increased in T2D: membrane transport of sugars, branched-chain amino acid (BCAA) transport, methane metabolism, xenobiotics degradation and metabolism, and sulphate reduction Increased in control: bacterial chemotaxis, flagellar assembly, butyrate biosynthesis, and metabolism of cofactors and vitamins.	[23]
53 T2D 49 IGT 43 Controls (Swedish)	69–72	Women (145)	Metagenomic sequencing	Increased in T2D: *Clostridium clostridioforme*, former-*Lactobacillus gasseri*, and *Streptococcus mutans* Decreased in T2D: *Roseburia*, *Clostridium spp*., *Eubacterium eligens*, *Coriobacteriaceae,* and *Bacteroides intestinalis*.	Increased in T2D: starch and glucose metabolism, fructose and mannose metabolism, ABC transporters for amino acids, ions and simple sugars, fatty acid biosynthesis, and cysteine and methionine metabolism Increased in control: flagellar assembly, and riboflavin metabolism.	[25]
13 T2D 64 Prediabetes 44 Controls (Chinese)	52–55	Not available	16S rRNA V3-V5 region	Increased in T2D: *Clostridiales*, *Dorea*, *Prevotella*, *Collinsella*, and *Ruminococcus* Decreased in T2D: *Bacteroides*, *A*. *muciniphila*, *F*. *prausnitzii*, *Haemophilus parainfluenzae*, and *Roseburia*	-	[26]
75 T2M 291 Controls (Danish)	50–66	Women (187) Men (179)	Metagenomic sequencing	Increased in T2D: *Prevotella copri* and *Bacteroides vulgatus* Decreased in T2D: *Roseburia*, *Bifidobacterium*, *Faecalibacterium*, *Oscillibacter*, *Coprococcus*, and *Butyrivibrio*	Increased in T2D: lipopolysaccharide and BCAA biosynthesis Decreased in T2D: BCAA transport into bacterial cells, methanogenesis, and pyruvate oxidation.	[27]
22 T1D 23 T2D 23 Controls (Polish)	20–65	Women (40) Men (28)	16S rRNA	Increased in T2D: *Ruminococcus*, *Enterobacteriaceae*, and *Verrucomicrobia* Decreased in T2D: *Bacteroides*, *Roseburia*, and *Faecalibacterium* (n.s.)	-	[28]
20 T2D 40 Controls (Chinese)	20–60	Women (42) Men (18)	16S rRNA V4-V5 region	Increased in T2D: *Dorea*, *Fusobacterium*, and *F*. *prausnitzii* Decreased in T2D: *Parabacteroides*, *Akkermansia*, *Bifidobacterium*, and *Streptococcus*	Increased in T2D: butyrate production via transferase, methanol conversion, and pentose phosphate pathway Decreased in T2D: tyrosine degradation, leucine degradation, and anaerobic fatty acid beta-oxidation	[29]
98 T2D 193 Controls (Africans)	41–70	Not available	16S rRNA V4 region	Increased in T2D: *Desulfovibrio piger*, *Prevotella*, *Eubacterium*, and *Peptostreptococcus* Decreased in T2D: *Collinsella*, *Ruminococcus lactaris*, *Anaerostipes*, *Epulopiscium*, and *Clostridium*	Increased in T2D: proteasome pathway Decreased in T2D: none	[21]
134 T2D 37 Controls (Chinese)	45–67	Women (92) Men (79)	16S rRNA V3-V4 region	Increased in T2D: *Prevotella*, *Dialister*, *and Sutterella* Decreased in T2D: *Bacteroides*, *Bifidobacterium*, *Clostridium XIVa*, *Parabacteroides*, *Staphylococcus*, *Granulicatella*, *Porphyromonas*, *Clostridium XI*, *Blautia*, *Anaerostipes*, *Clostridium XVIII*, *Fusicatenibacter*, *Enterococcus*, *Clostridium IV*, *Eggerthella*, and *Flavonifractor*.	-	[30]
46 T2D 75 CGI 178 IGT 189 IFG 523 Controls (Swedish)	57–61	Women (568) Men (443)	Metagenomic sequencing	Increased in T2D: *Coprococcus eutactus*, *Clostridiales bacterium*, and *Lachnospiraceae bacterium* Decreased in T2D: *Clostridium* sp., *C*. *hathewayi*, *Clostridium bolteae*, *C*. *symbiosum*, and *Roseburia faecis*	Increased in T2D: two-component systems, phosphotransferase systems, fructose and mannose metabolism, pentose phosphate pathway, bacterial biosynthesis of branched-chain amino acids, and metabolism of the B-group vitamins biotin and thiamine. Decreased in T2D: bacterial methanogenesis, glycolysis, peptidoglycan biosynthesis, vancomycin resistance, and DNA replication and transcription.	[22]

Abbreviations: y: years; n.s.: not significant. Bacteria: *A*. *muciniphila*: *Akkermansia muciniphila*; *C*. *hathewayi*: *Clostridium hathewayi*; *C*. *symbiosum*: *Clostridium symbiosum*; *F*. *prausnitzii*: *Faecalibacterium prausnitzii*.

**Table 2:** Main microbial metabolites related to T2DM.

Metabolite	Producing Bacteria (Genus or Species)	Mechanism of T2DM Risk	Reference
SCFA (acetate, propionate, and butyrate)	*Akkermansia*, *Ruminococcus*, *Faecalibacterium prausnitzii*, *Eubacterium*, *Roseburia*, *Blautia*, *Coprococcus*, *Anaerostipes*, and others	- Increases epithelial barrier function by regulation of TJP; - Reduces the passage of LPS, improving inflammation; - Stimulates the secretion of PYY and GLP-1 from L-cells in a GPR41 and GPR43 dependent manner; - Reduces appetite, insulin secretion, plasma glucose levels, and slow gastric emptying through stimulation of GLP-1 and GLP-2 secretion.	[39,40,41]
Imidazole propionate	Imidazole propionate: *Eggerthella lenta*, *Streptococcus mutans*, *Aerococcus urinae*, *Brevibacillus laterosporus*, and others	- Impairs glucose tolerance and insulin signaling by activating the p38γ-p62-mTORC1 pathway.	[42]
TMAO/TMA	*Desulfovibrio desulfuricans*, *Providencia*, *E*. *coli*, *Klebsiella pneumoniae*, *Sporosarcina*, and others.	- Exacerbates blockage of the insulin signaling pathway and promotes inflammation in adipose tissue.	[43]
Branched-chain amino acids	former-*Lactobacillus*, *Weissella*, *Leuconostoc, P. copri, and B. vulgatus*	- Promotes insulin resistance through serine phosphorylation of IRS-1; by persistent activation of mTORC1/S6K1.	[27,44,45]
Bile acids Secondary bile acids	Secondary bile acids: *Ruminococcus*, *Bifidobacterium*, *Bacteroides*, *Clostridium*, former-*Lactobacillus*, *Eubacterium*, *Listeria*, and others.	- Ligands of nuclear receptors, such as VDR, PXR, and FXR, induce TGR5 expression and regulate insulin and glucose sensitivity.	[46,47]
Tryptophan metabolites Tryptamine	*Clostridium bartlettii*, *Clostridium sporogenes*, *Ruminococcus gnavus*, *Bacteroides ovatus*, *Lactobaccilus acidophilus,Limosilactobacillus reuteri*, *Bifidobacterium fragilis, Bifidobacterium bifidum*, and others.	- Improves intestinal epithelial barrier function by the activation of PXR; - Stimulates insulin secretion, supresses appetite, and slows gastric emptying by stimulating GLP-1 secretion; - Promotes gastrointestinal motility by stimulating serotonin release; - Anti-inflammatory and anti-oxidative effects in the systemic circulation.	[48]

Abbreviations: SCFA: short chain fatty acids; TJP: tight junction proteins; LPS: lipopolysaccharides; PYY: peptide YY; GLP-1: glucagon-like peptide; GPR: G-protein coupled receptor; mTORC1: mechanistic target of rapamycin complex 1; S6K1: ribosomal S6 kinase 1; TMAO: trimethylamine-N-oxide; TMA: trimethylamine; BCAA: branched-chain amino acids; IRS-1: insulin receptor substrate 1; VDR: vitamin D3 receptor; PXR: pregnane X receptor; FXR: farnesoid X receptor; TGR5: G-protein-coupled bile acid receptor.

**Table 3:** Summary of clinical trials evaluating the effect of probiotic supplementation alone in individuals with T2DM.

Sample Size	Design	Duration	Intervention	Metabolic Outcomes	Microbiota Modifications	Functional Modifications	Study
Placebo (18) Intervention (16)	Single-blind clinical trial	6 weeks	Probiotic: 3000mg/day of *L*. *acidophilus*, *L*. *bulgaricus*, *L*. *bifidum,* and *L*. *casei*	Probiotic: n.s.	-	-	[77]
Placebo (53) Intervention (48)	Randomized, double-blind, parallel-group, controlled clinical trial	12 weeks	Probiotic: 3 × 10^10^ CFU/day of *L*. *acidophilus*, *L*. *casei*, *L*. *lactis*, *B*. *bifidum*, *B*. *longum*, and *B*. *infantis*	Probiotic: ↓ HbA1C, FI, HOMA-IR	Probiotic: ↑ *Bifidobacterium* spp., former-*Lactobacillus* spp.	-	[78]
Placebo (39) Intervention (39)	Randomized, single-centre, double-blind, placebo-controlled	12 weeks	Probiotic: 10^10^ CFU/day of *B*. *bifidum* W23, *B*. *lactis* W52, *L*. *acidophilus* W37, *L*. *brevis* W63, *L*. *casei* W56, *L*. *salivarius* W24, *L*. *lactis* W19, and *L*. *lactis* W58	Probiotic: ↓ HOMA-IR, FBG, Insulin, C-peptide, TG, LDL-c, WHR	-	-	[79]
Placebo (15) Intervention (29)	Randomized, double-blind, placebo-controlled trial	12 weeks	Probiotic: *L.* *reuteri* DSM 17938 LD: 10^8^ CFU/day HD: 10^10^ CFU/day	Probiotic: HD: ↑ ISI, DCA LD: ↑ unconjugated bile acids	Probiotic: ↑ *L. reuteri*	-	[80]
Placebo (22) Intervention (46)	Randomized, double-blind, placebo-controlled trial	9 months (6 month intervention)	Group 1: 4 × 10^9^ CFU/day of probiotic *L. reuteri* ADR-1 Group 2: 2 × 10^10^ CFU/day heat-killed *L. reuteri* ADR-3	Group 1: ↓ HbA1C, TC Group 2: ↓ SBP, IL-1β	Group 1: ↑ *L. reuteri* Group 2: ↑ *Bifidobacterium*	-	[81]
Placebo (22) Intervention (31)	Randomized, double-blind, single-centre, clinical trial	8 weeks	Probiotic: 1 sachet (10g)/day of 14 probiotic strains of former-*Lactobacillus* + *Lactococcus* (6 × 10^10^ CFU/g), *Bifidobacterium* (1 × 10^10^/g), *Propionibacterium* (3 × 10^10^/g), *Acetobacter* (1 × 10^6^/g) genera	Probiotic: ↓ HOMA-IR, TNF- α, IL-1β, WC	-	-	[82]
Placebo (30) Intervention (31)	Randomized, single-centre, double-blind, placebo-controlled clinical trial	6 months	Probiotic: 10^10^ CFU/day of *B*. *bifidum* W23, *B*. *lactis* W52, *L*. *acidophilus* W37, *L*. *brevis* W63, *L*. *casei* W56, *L*. *salivarius* W24, *L*. *lactis* W19 and *L*. *lactis* W58	Probiotic: ↓ HOMA-IR, FBG, Insulin, C-peptide, TG, TC, TC/HDL, CRP, TNF-α, IL-6, resistin, endotoxin ↑ adiponectin	-	-	[83]
Placebo (20) Intervention (20)	Randomized, parallel-group, placebo-controlled trial	8 weeks	Probiotic: 10^8^ CFU/day of *L*. *casei*	Probiotic: ↓ FBG, HOMA-IR, Insulin, fetuin-A, weight, BMI, WC ↑ SIRT1	-	-	[84]
Placebo (30) Intervention (30)	Randomized, double-blind, single-centre, placebo-controlled pilot trial	12 weeks	Probiotic: 2 × 10^11^ CFU/day of *L*. *plantarum* Lp-115, *L*. *bulgaricus* Lb-64, *L*. *gasseri* Lg-36, *B*. *breve* Bb-03, *B*. *animalis* sbsp. *lactis* Bi-07, *B*. *bifidum* Bb-06, *S*. *thermophilus* St-21, and *S*. *boulardii* DBVPG 6763	Probiotic: ↑ plasma butyrate Subgroup (metformin): ↓ FBG, HbA1C, insulin resistance, and zonulin ↑ plasma butyrate	Probiotic: n.s. beta diversity ↓ *P*. *copri*, *Flavonifractor plautii* ↑ *B*. *breve*, *B*. *caccae*, *Bacteroidales bacterium* ph8, *A*. *muciniphila*, *C*. *hathewayi* Subgroup (metformin): ↓ *Bactoides uniformis* ↑ *B*. *breve*, *B*. *caccae*, *Anaerotruncus colihominis*	Subgroup (metformin): pyruvate fermentation to butanoate, and *Bifidobacterium* shunt pathways	[85]

Abbreviations: N.s.: not significant; HbA1C: hemoglobin A1C; HOMA-IR: Homeostatic Model Assessment for Insulin Resistance; FBG: fasting blood glucose; IR: insulin resistance; TG: triglycerides; WHR: waist-to-hip ratio; LD: low dose; HD: high dose: ISI: insulin sensitivity index; DCA: deoxycholic acid; SBP: systolic blood pressure; IL-1β: interleukin 1 beta; IL-6: interleukin 6; TNF-α: tumor necrosis factor alpha; WC: waist circunference; TC: total cholesterol; LDL-c: low-density lipoprotein cholesterol; HDL: high density lipoprotein; CRP: C-reactive protein; BMI: body mass index; SIRT1: sirtuin 1. Bacteria: *A*. *muciniphila*: *Akkermansia muciniphila*; *B*. *caccae*: *Bacteroides caccae*; *B*. *bifidum*: *Bifidobacterium bifidum*; *B*. *breve*: *Bifidobacterium breve*; *B*. *longum*: *Bifidobacterium longum*; *B*. *infantis*: *Bifidobacterium infantis; C. hathewayi: Clostridium hathewayi*; *L*. *casei*: *Lacticaseibacillus casei*; *L. plantarum:* *Lactiplantibacillus plantarum;* *L*. *acidophilus*: *Lactobacillus acidophilus*; *L*. *bifidum*: *Lactobacillus bifidum*; *L*. *bulgaricus: Lactobacillus bulgaricus*; *L*. *gasseri*: *Lactobacillus gasseri*; *L*. *lactis*: *Lactococcus lactis*; *L*. *brevis*: *Levilactobacillus brevis; L. salivarius: Ligilactobacillus salivarius*; *L. reuteri: *Limosilactobacillus* reuteri; P*. *copri*: *Prevotella copri*; *S. boulardii: Saccharomyces boulardii*; *S. thermophilus: Streptococcus thermophilus*.
